# Enhanced soft magnetic properties and high-frequency stability of FeNiMo powder cores by coating SiO_2_ insulation layer†

**DOI:** 10.1039/d3ra01523h

**Published:** 2023-05-26

**Authors:** Jinming Lai, Likang Xiao, Zhengwei Xiong, Leiming Fang, Wenkun Zhu, Fangguang Kuang, Zhipeng Gao

**Affiliations:** a School of Microelectronics, Xidian University Xi'an 710071 China; b China Electronics Technology Group Corporation 29th Research Institute, Sichuan Broadband Microwave Circuit High Density Integration Engineering Research Center Chengdu 610036 China; c School of Electronic Science and Engineering, University of Electronic Science and Technology of China, Southwest Institute of Applied Magnetism Mianyang 621010 China; d Joint Laboratory for Extreme Conditions Matter Properties, School of Mathematics and Physics, Southwest University of Science and Technology, Sichuan Civil-military Integration Institute Mianyang 621010 China zw-xiong@swust.edu.cn; e Institute of Physics Nuclear and Chemistry, China Academy of Engineering Physics Mianyang 621900 China; f School of Physics and Electronic Information, Gannan Normal University Ganzhou 341000 China; g Institute of Fluid Physics, China Academy of Engineering Physics Mianyang 621900 China z.p.gao@foxmail.com

## Abstract

FeNiMo/SiO_2_ powder cores were prepared using the sol–gel method. Tetraethyl orthosilicate (TEOS) was added to produce an amorphous SiO_2_ coating outside the FeNiMo particles to form a core–shell structure. The thickness of the SiO_2_ layer was designed by varying the concentration of TEOS, and the optimized permeability and magnetic loss of the powder core could achieve 78.15 and 633.44 kW m^−3^ at 100 kHz and 100 mT, respectively. Compared with other soft magnetic composites, these FeNiMo/SiO_2_ powder cores have a significantly higher effective permeability and a lower core loss. Surprisingly, the high-frequency stability of permeability could be substantially enhanced through the insulation coating process in which *μ*_f_/*μ*_100 kHz_ could reach 98.7% at 1 MHz. In comparison with 60*μ* commercial products, the comprehensive soft magnetic properties of the FeNiMo/SiO_2_ cores were superior to most manufacturers, which would be potentially applied to high-performance inductance devices in high-frequency ranges.

## Abstract 2

The high-frequency stability of soft magnetic composites could be substantially enhanced through the insulation coating process in which *μ*_f_/*μ*_100 kHz_ could reach 98.7% at 1 MHz.

## Graphical abstract

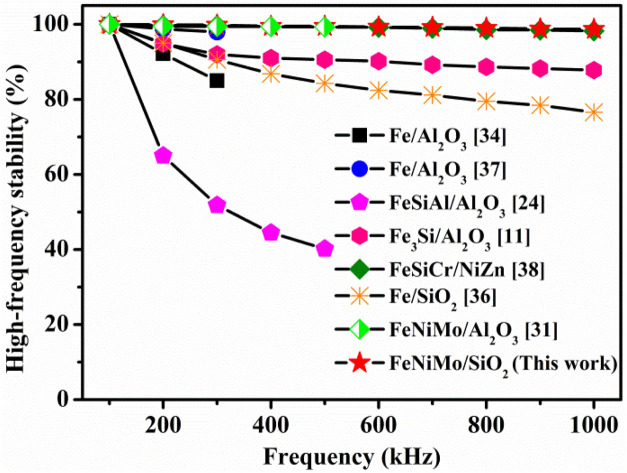

## 1. Introduction

Metal soft magnetic materials are widely used as inductors, transformers, and filters due to the high magnetic flux densities at saturation, high permeabilities, and low magnetostrictive coefficients.^1^ Nowadays, the common metal soft magnetic materials are Fe, FeSiAl, FeSiCr, FeNi, and FeNiMo. By comparison, FeNiMo possesses higher permeability and lower eddy current loss than that of other metal soft magnetic materials,^2,3^ which can be applied to higher operating frequencies. Unfortunately, the low resistivity of metal soft magnetic materials leads to large eddy current loss, and further performance decreases significantly with the increase of temperatures, limiting its application at high frequencies. Soft magnetic composites (SMCs) with high resistivity and low eddy current loss were developed by coating a layer of insulation material on the surface of the soft magnetic particles.^4,5^ There are two types of insulation coating materials, including organic and inorganic materials.^6–9^ Wherein, the inorganic materials are more popular owing to the poorer stability and easier decomposition of organic materials at high temperatures.^5,7^ As a typical representative of inorganic materials, SiO_2_ is widely utilized because of its simple preparation process, easy synthesis, excellent insulation properties, and heat resistance.

SiO_2_ is typically coated with silane coupling agent (APTES) and tetraethyl orthosilicate (TEOS) using the sol–gel method. As the most important silicon source, TEOS can hydrolyze to produce SiO_2_, and then generate SiO_2_ coating outside the metal soft magnetic particles to form a core–shell structure. Li *et al.*^10^ fabricated Fe_85_Si_9.6_Al_5.4_/SiO_2_ SMCs by using the sol–gel method and adjusting the amounts of TEOS and APTES. The thickness of the SiO_2_ coating increases as the TEOS content rises. Fan *et al.*^11^ prepared FeSiAl/SiO_2_ SMCs by tuning the amount of TEOS by the sol–gel method. It has been confirmed that, as the TEOS content rises, the denser the powder core becomes, the thicker is the coating layer on the surface of the powder. Yang *et al.*^12^ fabricated Fe/SiO_2_ SMCs using the sol–gel method, which revealed that a uniform SiO_2_ layer was formed on the surface of Fe powders, significantly reducing the loss. Zhou *et al.*^13^ prepared soft magnetic particle cores from FeSiBCr/SiO_2_ using the sol–gel method. The results show that the samples with the optimal thickness have excellent magnetic properties. The excessively thick coating increases the iron core's hysteresis loss, resulting in an increase in total loss.^13^ As mentioned above, controlling the amount of TEOS and APTES can determine the thickness of the SiO_2_ coating, the shape of the core–shell structures, and ultimately the magnetic properties of the samples. To date, few studies on FeNiMo cores with the insulation coating of SiO_2_ have been conducted. In addition, FeNiMo in nature has a higher frequency stability of permeability relative to other metal soft magnetic materials. The SiO_2_ coating is further expected to enhance the high-frequency stability of FeNiMo.

In this work, the soft magnetic FeNiMo/SiO_2_ composites were successfully prepared by the sol–gel method. The coating thickness of the samples was modified by adjusting the amount of TEOS. The sample's magnetic properties as well as the hysteresis loss and eddy current loss were studied systematically.

## 2. Experimental procedure

### 2.1 Sample preparation

FeNiMo powders with an average particle size of 22.3 μm were obtained from a commercial supplier (Beijing Compo Advanced Technology Co., Ltd.). Their Ni and Mo contents were 86.67 wt% and 2.27 wt%, respectively, and the Fe content was 10.64 wt%. Triethoxysilane C_9_H_23_NO_3_Si (APTES, Chron Chemicals) and tetraethyl orthosilicate C_8_H_20_O_4_Si (TEOS, Chron Chemicals) were used as coupling agents and silicon sources, respectively. During the molding process, zinc stearate was used as a lubricant, and epoxy resin as an adhesive.

20 grams of FeNiMo powder was used with 170 mL of absolute ethanol, and placed in a water bath. A mixture of 5 mL of APTES and 30 mL of deionized water was then added to the solution, and the mixture was maintained under mechanical stirring at 500 rpm for 1 hour. Following the APTES reaction for 1 hour at room temperature, TEOS was added and the temperature was further raised to 50 °C. After heating the solution for three hours, FeNiMo/SiO_2_ powder was obtained. The coated powder was dried at 60 °C for one hour.

Epoxy resin (1 wt%) was dissolved in acetone, then the coated powder was added and stirred until the acetone was evaporated. As a lubricant, 0.25 wt% zinc stearate was added to the coated powder before pressing. The powder mixture was pressed into an annular sample with an outer diameter of 10.2 mm and an inner diameter of 5.08 mm under a pressure of 1950 MPa. Following annealing at 600 °C for 1 hour in an argon atmosphere, the sample was cooled in the furnace. The parameters of preparation FeNiMo/SiO_2_-insulated powders and SMCs were optimized in the previous work.^14^

### 2.2 Material characterization

The particle sizes were measured by a laser particle size analyzer (Malvern Mastersizer 3000). The microstructure of FeNiMo powder and coated powder was obtained by scanning electron microscopy (SEM, TM4000, Hitachi). Energy dispersive spectroscopy (EDS) elemental mapping was used to characterize the SiO_2_ insulating coating. The density of the prepared sample was calculated using a water boiling method based on the Archimedes principle. The electrical switching behavior of the films was studied by using the four-probe system in dismal surroundings, which was equipped with a Keithley 2400 (Tektronix, America) source meter. X-ray diffraction was used to determine the phase structure of the powder (XRD, SmartLab, Rigaku). The density of SMCs was determined using the density balance. AC magnetic properties of the samples were measured using a *B*–*H* curve analyzer (SY-8218, Iwatsu) at a frequency range of 10–1000 kHz and a *B* value of 100 mT, that is, under typical industrial conditions for FeNiMo soft magnetic products. Based on the core loss *P*_cv_, hysteresis loss *P*_h_ and eddy current loss *P*_e_ are obtained.^15,16^ In order to determine the effective permeability, the inductance of the magnetic particle core was measured using an impedance analyzer:^11^1
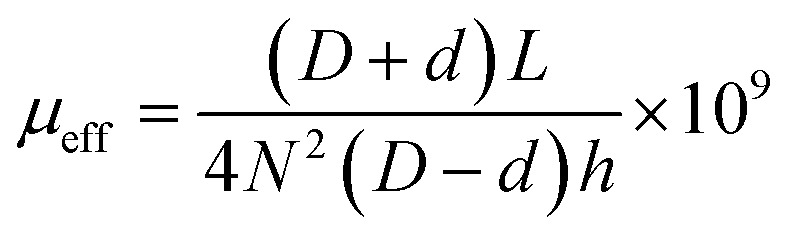
where *D*, *d*, and *h* are the outer diameter, inner diameter, and the height of the magnetic particle core, respectively. *N* is the number of turns in the coil, and *L* is the inductance.

## 3. Results and discussion

### 3.1 Microstructure

**Fig. 1:**
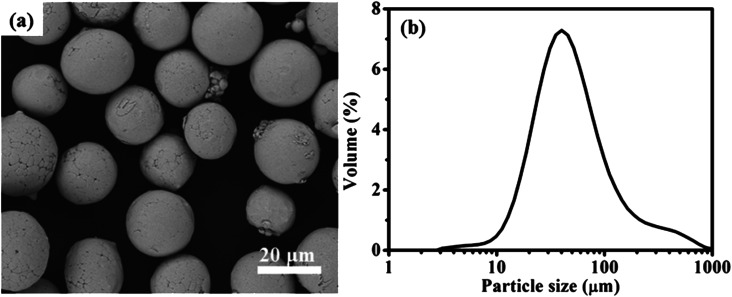
(a) SEM image and (b) particle size distribution curve of FeNiMo powders.

Fig. 1(a) shows the SEM image of FeNiMo powder, which presents an obvious spherical structure without agglomeration. The normal distribution diagram of the powder particle sizes obtained by the laser particle size analyzer is shown in Fig. 1(b), and the average particle size is 22.3 μm. By laser particle size analyzer, the average sizes of FeNiMo/SiO_2_ powder cores with different TEOS volumes can be obtained, as shown in Fig. S1.† Obviously, the particle sizes are increasing with the increase in TEOS concentrations. Compared with pure FeNiMo particles (Fig. 1(b)), the thickness of the SiO_2_ layer can be deduced in the range of 900 nm to 4.7 μm.

**Fig. 2:**
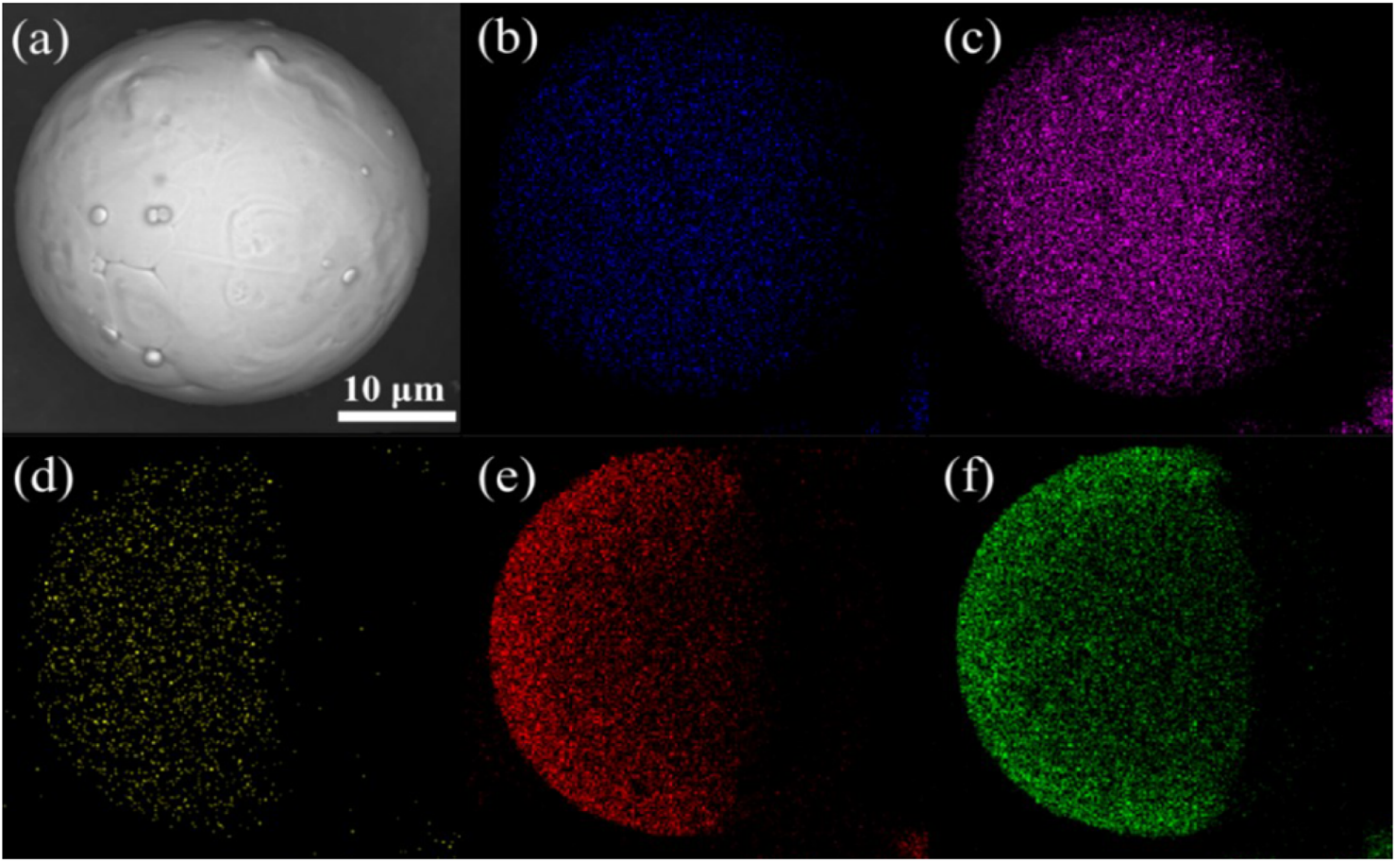
(a) SEM images of the FeNiMo powders coated with SiO_2_ and corresponding EDS elemental mapping of (b) Fe, (c) Ni, (d) Mo, (e) O, and (f) Si.

After the FeNiMo powders were coated with the SiO_2_ layer by the sol–gel method, the SEM (Fig. 2(a)) and EDS elemental mapping of the corresponding area (Fig. 2(b)–(f)) showed that the coated sample was mainly composed of Fe, Ni, Mo, O, and Si elements. In addition, the elements were evenly distributed, and O and Si elements showed the same mapping intensity in the same bright area. The elemental distribution maps prove that the O and Si were successfully distributed uniformly on the particle surface.

**Fig. 3:**
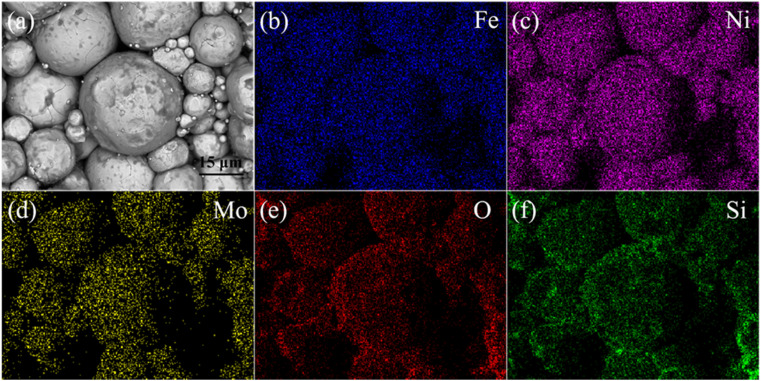
(a) Sectional SEM and (b–f) EDS elemental distribution of FeNiMo/SiO_2_ powder cores annealed at 600 °C.

Fig. 3 shows the sectional SEM image and corresponding element distribution of FeNiMo/SiO_2_ powder cores annealed at 600 °C. From Fig. 3(a), it can be seen that the SiO_2_ coating is basically intact without the melting phenomenon after the pressed powder core was annealed at 600 °C. As shown in Fig. 3(b)–(f), Si and O elements were uniformly distributed in the FeNiMo particles, and there were no elements in the gaps between particles, proving that the core–shell structure of the sample was not damaged at 600 °C. In this case, the diffusion of the atoms that compose the insulating layer (Si and O) into the ferromagnetic FeNiMo phase can be almost negligible. Our past works have demonstrated that the excessive annealing temperatures led to gradual damage of the insulating layer, and further resulted in reduced resistivity and poor frequency characteristics.^14^ Several previous investigations also reported that the amorphous SiO_2_ layer could be well coated with FeSi and carbonyl iron particles after high-pressure pressing and high-temperature annealing.^17,18^

### 3.2 Phase structure

**Fig. 4:**
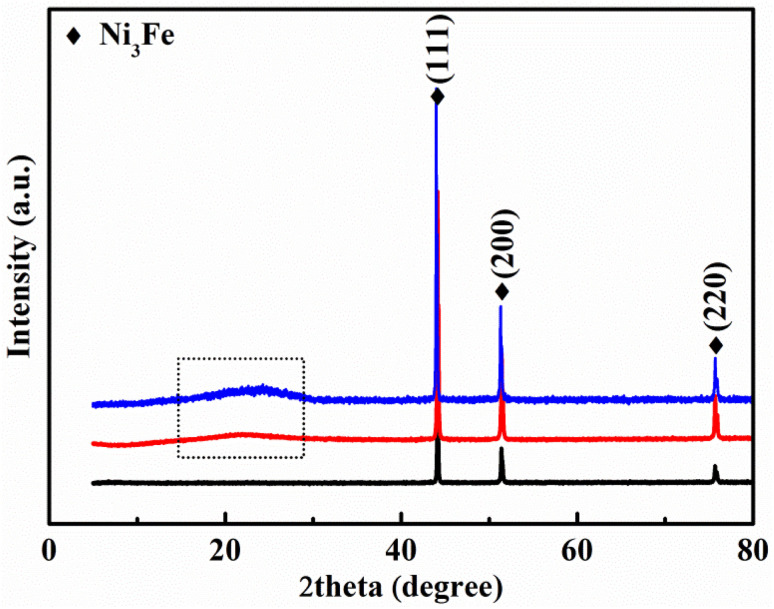
XRD patterns of raw FeNiMo powders (black), FeNiMo/SiO_2_ powders (red), and FeNiMo/SiO_2_ SMCs after annealing at 600 °C (blue).

Fig. 4 shows the XRD patterns. The diffraction peaks of raw powders are consistent with the indexed peaks of the Ni_3_Fe crystal structure (JCPDS card no. 88-1715). The raw FeNiMo powders have only a Ni_3_Fe crystalline phase within the accuracy of the instrument. With the coated SiO_2_ insulation, there is also an amorphous SiO_2_ phase at 15°–30°. Significantly, the amorphous SiO_2_ bulge still existed in the FeNiMo/SiO_2_ powder cores, indicating that the coated SiO_2_ layer was not damaged after annealing at 600 °C. The elemental ratios of raw FeNiMo and FeNiMo/SiO_2_ powders were obtained from the EDS spectrum, as shown in Table S1.† After the chemical deposition process, the Si and O concentrations were significantly increased. To confirm the core–shell structure, we further re-tested the EDS elemental distribution mapping of FeNiMo/SiO_2_ powders. Accordingly, to select the incompletely coated particles (Fig. S2†), the core–shell structures were clearly observed. Combined with the EDS elemental mapping map (Fig. 2), elemental ratios (Table S1†), and XRD analysis, it can be concluded that the FeNiMo powders were successfully coated with a uniform SiO_2_ layer by the sol–gel method, forming the core–shell FeNiMo/SiO_2_ SMCs.

### 3.3 Soft magnetic properties

According to the theory, the core losses (*P*_cv_) are made up of hysteresis loss (*P*_h_), eddy current loss (*P*_e_), and residual loss (*P*_r_). At low magnetic induction, *P*_r_ is negligible. As a result, core losses can be expressed as follows:^19,20^2
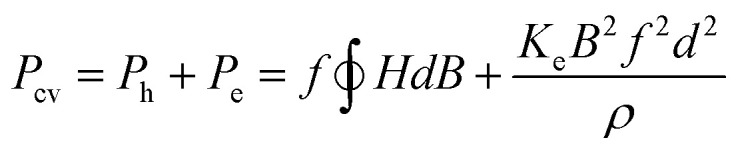
where *f*, *H*, and *B* are the frequency, magnitude of the magnetic field, and magnetic induction strength used in sample testing, respectively. *K*_e_ is a constant. *d* and *ρ* are the particle size and resistivity, respectively. *P*_h_ is the energy consumed by ferromagnets during repeated magnetization due to hysteresis. The influence of hysteresis loss is mainly caused by the pinning effect of the magnetic domain wall during magnetization, which is mainly caused by the stress, dislocations, defects, and pores formed by the compression. Here, high-temperature annealing can reduce internal stress, dislocation, and defect density, thereby improving the magnetic properties of the magnetic powder cores. Eddy current loss increases with the increase of the frequencies and decreases with the increase in the electrical resistivity. Thus, the total core loss of a magnetic device is the sum of the eddy current losses and hysteresis losses.

**Fig. 5:**
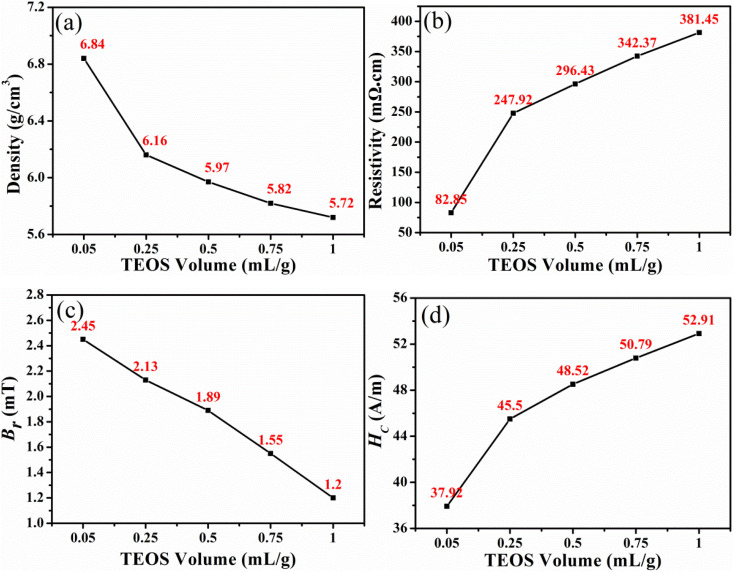
Effect of the addition of TEOS on (a) density, (b) resistivity, (c) residual magnetic flux density (*B*_r_), and (d) coercivity (*H*_c_) of the FeNiMo/SiO_2_ SMCs at various frequencies and *B* = 100 mT.

The density and resistivity of the FeNiMo/SiO_2_ powder cores prepared with different amounts of TEOS are shown in Fig. 5(a) and (b). With the increase in the TEOS amount, the density is decreasing and the resistivity is increasing. Combined with SEM and XRD results, it can be deduced that the SiO_2_ coating becomes thicker with the more TEOS amount. The more insulating SiO_2_ shell on the FeNiMo core induces increased resistivity, which is a direct inducement to reduce the eddy currents. The increase of non-magnetic SiO_2_ could further result in the decrease of *B*_r_ (Fig. 5(c)), meaning the weakening of magnetic properties. Meanwhile, *H*_c_ is gradually increased as the TEOS content increased (Fig. 5(d)). The enhanced *H*_c_ can directly cause an increase in hysteresis loss. The presence of the nonmagnetic SiO_2_ insulating layer between the magnetic FeNiMo particles acts as an air gap, serving as a demagnetizing field. The presence of pores or a non-magnetic SiO_2_ immobilizes the domain walls and increases the energy requirement for completing the same magnetization or demagnetizing process.^21,22^ Therefore, the *H*_c_ for FeNiMo/SiO_2_ composite compact is higher than that of FeNiMo compacts.

**Fig. 6:**
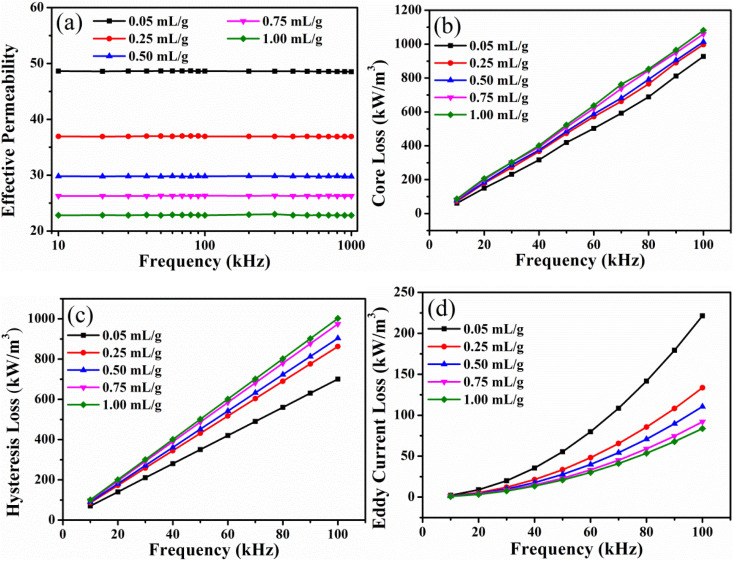
Effect of the addition of TEOS on AC magnetic properties of the FeNiMo/SiO_2_ SMCs at various frequencies and *B* = 100 mT: (a) effective permeability, (b) core loss, (c) hysteresis loss, (d) eddy current loss.

The AC magnetic properties of SMCs manufactured with the addition of the TEOS at various frequencies and *B* = 100 mT are depicted in Fig. 6. The effective permeability of the SMCs prepared at different TEOS concentrations remains unchanged with the increase of frequencies, and the change rate was less than 1% in the range of 10–1000 KHz. As the concentration of TEOS increases, the effective permeability gradually decreases. The effective permeability was optimized as 48.6 obtained with TEOS concentration of 0.05 mL g^−1^ (100 kHz, 100 mT). The SiO_2_ insulation coating is primarily responsible for the decreased effective permeability. As shown in Fig. 6(b), the core loss of SMCs increases as the TEOS concentration rises. The total sample loss is lowest as the TEOS concentration is 0.05 mL g^−1^, with a value of 926.94 kW m^−3^ (100 kHz, 100 mT). The sample's *P*_h_ increases as the TEOS concentration rises (Fig. 6(c)). The thick SiO_2_ insulation layer results in a high fraction of the non-magnetic phase,^13^ and the increased number of particle walls. The equivalent air gaps for the SiO_2_ can act as the pinning center for the domain wall to hinder the movement of the magnetic domain. Thus, a larger number of pinning centres are created by increasing the thickness of the SiO_2_ layer, leading to a higher *P*_h_. Among them, the *P*_h_ at the TEOS concentration of 0.05 mL g^−1^ is 700.63 kW m^−3^ (100 kHz, 100 mT). Fig. 6(d) demonstrates that the *P*_e_ value decreases as the TEOS concentration increases, attributing to the increased resistivity of SMCs as a result of an increase in SiO_2_. The increase in the core loss of SMCs is attributable to the fact that the increase in the *P*_h_ exceeds the decrease in *P*_e_. The dominant role of *P*_h_ in the total losses demonstrates that the magnetic mechanism can be attributed to the pinning effect of the domain wall. While the TEOS concentration is 0.05 mL g^−1^, the SMCs at 100 kHz and 100 mT have the highest effective permeability (48.6) and the lowest loss (926.94 kW m^−3^). The greater the amount of non-magnetic substances, the more the magnetism is diluted, resulting in a lower effective permeability.^23,24^ Additionally, the sample with a higher SiO_2_ content exhibited a lower density because it possessed more air gaps, a higher non-magnetic SiO_2_ content, and a higher inner demagnetizing field,^18^ which resulted in the larger *H*_c_ and lower magnetic induction values. Consequently, as the density increased, the coercivity decreased, and the hysteresis loss decreased.^25^ Apparently, the increased *P*_h_ arising from the domain wall pinning outweighed the decrease of *P*_e_, resulting in an overall increased *P*_cv_. This explains why the samples exhibited decreased *μ*_e_ and increased *P*_cv_ with increasing TEOS concentrations.

**Fig. 7:**
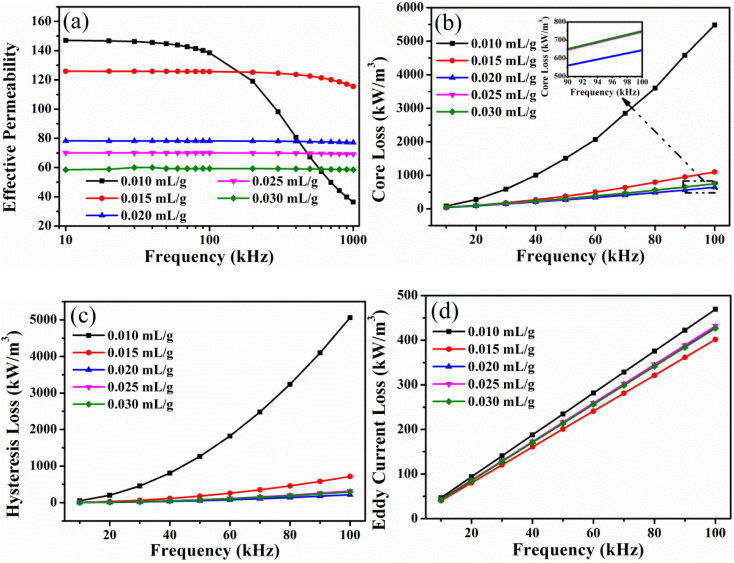
Effect of small concentration of TEOS on AC magnetic properties of the FeNiMo/SiO_2_ SMCs at various frequencies and *B* = 100 mT: (a) effective permeability, (b) core loss, (c) hysteresis loss, (d) eddy current loss.

**Fig. 8:**
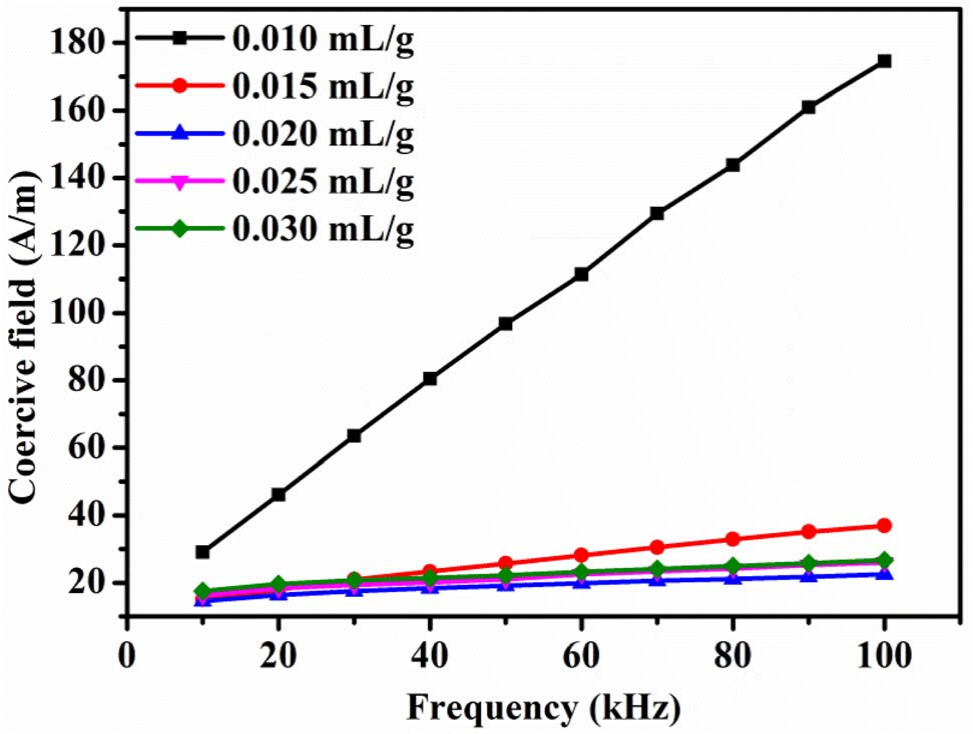
Effect of the TEOS concentration on coercive field of the FeNiMo/SiO_2_ SMCs at various frequencies and *B* = 100 mT.

In order to optimize the magnetic properties, the FeNiMo/SiO_2_ SMCs with lower TEOS concentrations were prepared, as shown in Fig. 7. The effective permeability of SMCs decreases gradually with increasing the TEOS concentration (Fig. 7(a)). Moreover, the high-frequency stability of SMCs was enhanced. In the frequency range of 100–1000 kHz, the effective permeability of the samples with a TEOS concentration of 0.02 mL g^−1^ decreases slightly with the increase of frequencies, and the change rate was less than 3%. Fig. 7(b) displays the core loss of SMCs manufactured with varying concentrations of TEOS. As the concentration of the TEOS increases, the core loss of the sample decreases and then rises until it reaches a minimum at the TEOS concentration of 0.02 mL g^−1^. As the TEOS concentration increases, the sample's hysteresis loss first decreases and then increases, reaching a minimum at 0.02 mL g^−1^ (Fig. 7(c)). Loss of hysteresis is proportional to coercivity (Fig. 8). Fig. 7(d) demonstrates that the sample's eddy current loss reaches its minimum at the TEOS concentration of 0.015 mL g^−1^. Eddy current loss is closely related to the sample's resistivity. The coated SiO_2_ insulating layer could effectively increase the distance between magnetic particles and restrict the eddy current between the particles. Meanwhile, a relatively thick insulating layer played a satisfactory insulating role and effectively reduced *P*_e_. However, this *P*_h_ increases slightly in the 0.025 and 0.03 mL g^−1^ samples, which affects the increase of *P*_e_ once the coating layer proceeds beyond a certain thickness limit.^26^ Consequently, the eddy current loss of SMCs was slightly altered. Therefore, as 0.02 mL g^−1^ TEOS was added to FeNiMo/SiO_2_ SMCs, the magnetic properties were the best. The effective permeability was stable and reached the highest value of 78.15 and the lowest core loss of 633.44 kW m^−3^ at 100 kHz and 100 mT.

FeNiMo/SiO_2_ SMCs exhibited a coercive field in Fig. 8. The coercive field of the sample increases monotonously with the increase of frequencies. The change law of coercivity is the same as the hysteresis loss. The coercive field is sharply reduced when TEOS was added at 0.015 mL g^−1^ or more. Lower coercivity means a smaller hysteresis loop area and correspondingly smaller hysteresis loss and *vice versa* because hysteresis loss is closely related to the coercivity and hysteresis loop area.^27^ By reducing the coercive field, the hysteresis loss of the sample was reduced, which in turn reduced its core loss.

**Fig. 9:**
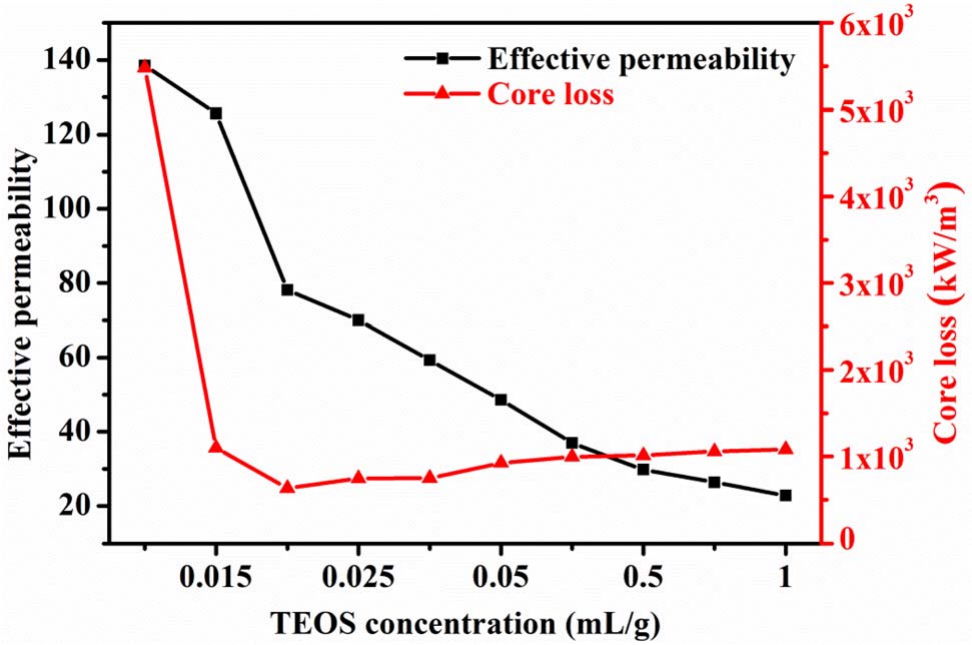
Magnetic properties of SMCs prepared with different TEOS concentrations measured at 100 mT.

The effective permeability and core loss of SMCs prepared in the concentration range of 0.01–1 mL g^−1^ TEOS are shown in Fig. 9. As the concentration of TEOS rises, the effective permeability of SMCs decreases gradually. The non-magnetic SiO_2_ phase in SMCs is responsible for a reduction in effective permeability.^28^ An increase in the distance between ferromagnetic particles could result in a reduction in their effective permeability. The increasing thickness of the SiO_2_ layer could increase the distance between magnetic particles, resulting in a slower magnetic response of the magnetic particles to an applied magnetic field and a consequent increase in *H*_c_.^11^ This causes the hysteresis loss of SMCs to increase. A sufficient amount of SiO_2_ can reduce eddy current loss significantly. Considering the overall magnetic properties of the SMCs, the sample with a TEOS content of 0.02 mL g^−1^ shows the optimum performance properties with an effective permeability of 78.15 and core loss of 633.44 kW m^−3^ at 100 kHz and 100 mT.

Table 1 displays the effective permeability and loss results for various types of soft magnetic composites. In our past works, we demonstrated that the pure FeNiMo compacts presented higher effective permeability (152) and larger loss (7038 kW m^−3^) after 600 °C pretreatment process in H_2_/Ar mixture.^14^ The high-temperature treatment in a reducing atmosphere could effectively remove metal oxides from the FeNiMo material surface and increase the content of elemental states, thereby further significantly improving the effective permeability of FeNiMo raw powders. Kollár also reported that the pure FeNiMo with smoothing the surfaces of individual particles exhibited smaller hysteresis loss and larger eddy loss.^29,30^ At 100 kHz and 100 mT, the effective permeability and the core loss of FeNiMo/SiO_2_ SMCs optimized by the insulation coating process were 78.15 and 633.44 kW m^−3^, respectively. Obviously, the SiO_2_ coating effectively reduces the core loss of FeNiMo. In comparison to FeNiMo-based SMCs, our samples have a greater effective permeability and lower core loss. Relative to Fe-based SMCs coated with SiO_2_, Al_2_O_3,_ and NiZn ferrite, the FeNiMo/SiO_2_ SMCs have a significantly higher effective permeability and a significantly lower core loss. Comparative analysis revealed that FeNiMo/SiO_2_ SMCs have higher effective permeability and lower core loss than the majority of the soft magnetic particle cores under identical test conditions.

**Table 1:** Comparison of magnetic properties of different types of SMCs

SMCs	50 kHz		100 kHz		*B*/mT	Ref.
	*μ*_eff_	*P*_cv_/kW m^−3^	*μ*_eff_	*P*_cv_/kW m^−3^		
FeNiMo/SiO_2_	78.17	6.0664	78.15	24.318	20	This work
		52.759		133.48	50	
		263.29		633.44	100	
FeNiMo/Al_2_O_3_	87.6	321.78	—	—	100	31
FeNiMo/resin	—	—	30	6180	100	32
Fe/Al_2_O_3_	88.1	310.65	—	—	20	33
Fe/Al_2_O_3_	—	—	42	353	20	34
Fe/NiZn	43.5	94.83	43.14	199.3	20	35
Fe/SiO_2_/MnZn	—	—	64	104.9	20	36
Fe_3_Si/Al_2_O_3_	113.9	3609	—	—	50	11

### 3.4 High-frequency stability

**Fig. 10:**
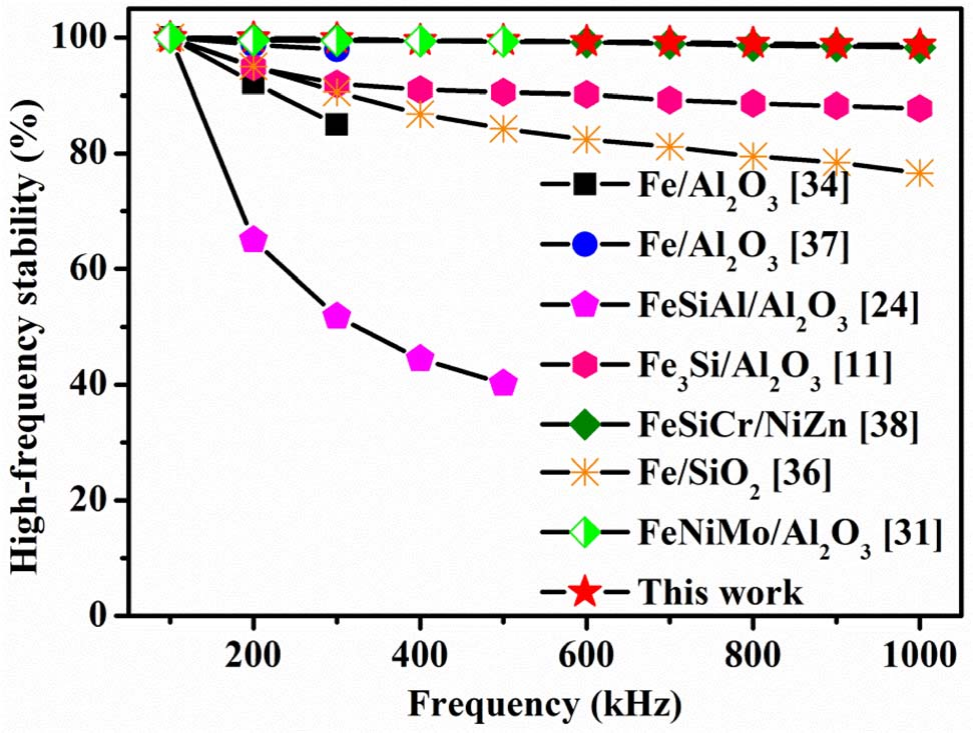
The comparison of high-frequency stability for different types of SMCs.

The index parameter of high-frequency stability in the range of 100 kHz to 1 MHz, *μ*_f_/*μ*_100 kHz_ was used, where *f* represents the effective permeability at the given frequency and *μ*_100 kHz_ is the effective permeability at 100 kHz. Fig. 10 shows a comparison of the high-frequency stability for various SMCs. In this study, the high-frequency stability of the optimized powder cores was significantly enhanced through the insulation coating process. Here 1 MHz stability is achieved at 98.7%. In comparison to other SMCs such as FeNiMo/Al_2_O_3_,^31^ Fe/Al_2_O_3_,^34,37^ Fe_3_Si/Al_2_O_3_,^11^ FeSiAl/Al_2_O_3_,^24^ Fe/SiO_2_ (ref. 36) and FeSiCr/NiZn,^38^ the FeNiMo/SiO_2_ cores have enhanced high-frequency stability in the range of 100–1000 kHz.

### 3.5 Commercial product comparison

**Fig. 11:**
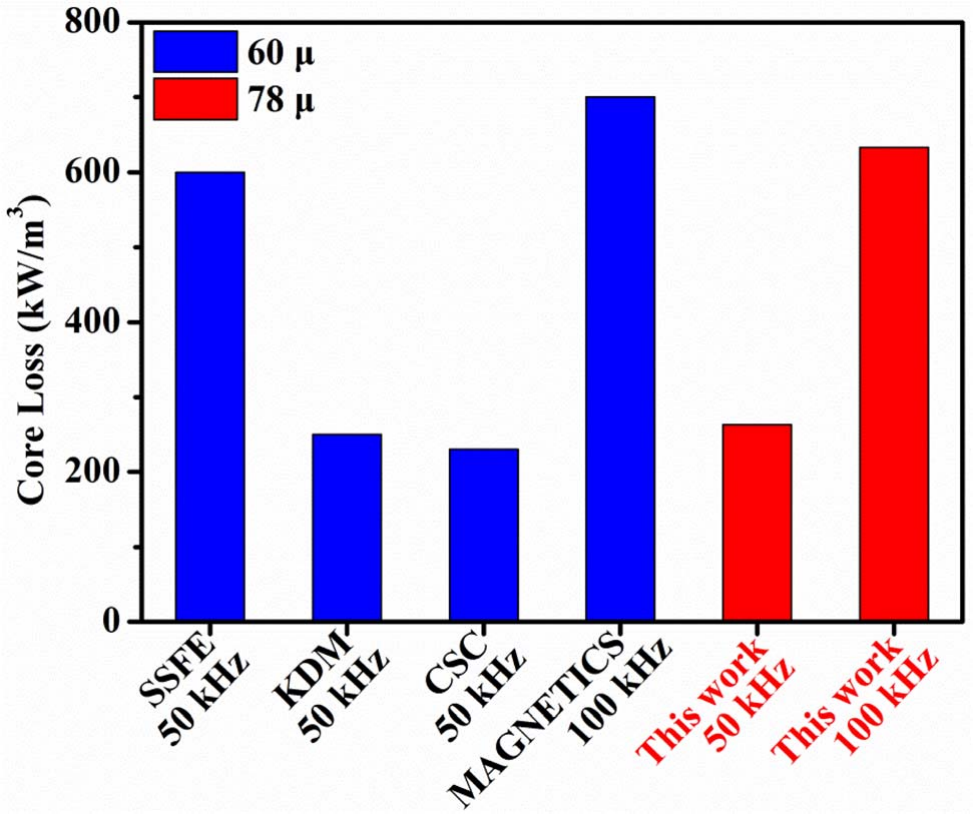
Comparison of magnetic properties with domestic and foreign commercial MPP cores at 100 mT.

Fig. 11 illustrates the results of the comparison with the magnetic properties of domestic and foreign commercial molypermalloy powder (MPP) cores at 100 mT. More specific data are presented in Table S2 in the ESI.† In comparison with 60*μ* commercial products, the samples prepared in this paper are superior to those of Beijing Seven Star Flight Electron Co., Ltd. (abbreviated as SSFE) and Magnetics. Furthermore, the magnetic properties of our samples are similar to those of CSC and KDM. In addition, the samples prepared in this study have a higher effective permeability of 78μ.

## 4. Conclusions

The magnetic properties of the sol–gel method-prepared FeNiMo/SiO_2_ soft magnetic composites were investigated. The results indicate that a uniform SiO_2_ insulating layer can effectively separate FeNiMo alloy particles. By adding TEOS, the thickness of the SiO_2_ insulating layer can be precisely controlled. However, excessive TEOS could decrease effective permeability and increase hysteresis loss in SMCs. This causes the magnetic properties of SMCs to decrease. The effective permeability of the SMCs decreases as the TEOS concentration range increases from 0.01 to 1 mL g^−1^, whereas the core loss initially decreases and then increases. Specifically, FeNiMo/SiO_2_ SMCs compacted with TEOS concentration of 0.02 mL g^−1^ exhibit favorable performance characteristics, including good frequency stability, high effective permeability (78.15 at 100 kHz), and low core loss (633.44 kW m^−3^ at 100 kHz and 100 mT). The SMCs described in this paper have higher effective permeability, lower loss, and enhanced high-frequency stability compared to the results reported in the literature. The magnetic properties of SMCs are comparable to those of commercially available materials. This research is anticipated to serve as a valuable resource for the domestic development of high-frequency SMCs.

## Conflicts of interest

There are no conflicts to declare.

## Supplementary Material

RA-013-D3RA01523H-s001
