# Supplementary material for: Enhanced soft magnetic properties and high-frequency stability of FeNiMo powder cores by coating SiO2 insulation layer
Source: RSC Adv. 2023 May 26;13(23):15892–900. doi: 10.1039/d3ra01523h (PMC10214003; doi:10.1039/d3ra01523h)
Supplement: RA-013-D3RA01523H-s001 [file RA-013-D3RA01523H-s001.pdf]

Electronic Supplementary Material (ESI) for RSC Advances.
This journal is © The Royal Society of Chemistry 2023

Electronic supplementary information

Enhanced soft magnetic properties and high-frequency stability of FeNiMo powder cores by coating SiO_2 insulation layer

Jinming Lai[1, †], Likang Xiao[2, †], Zhengwei Xiong[3, *], Leiming Fang[4], Wenkun Zhu[3], Fangguang Kuang[5], Zhipeng Gao[6, *]

[1] School of Microelectronics, Xidian University, Xi'an 710071; China Electronics Technology Group Corporation 29th Research Institute; Sichuan Broadband Microwave Circuit High Density Integration Engineering Research Center, Chengdu 610036, China

[2] School of Electronic Science and Engineering, University of Electronic Science and Technology of China; Southwest Institute of Applied Magnetism, Mianyang 621010, China

[3] Joint Laboratory for Extreme Conditions Matter Properties, School of Mathematics and Physicsm, Southwest University of Science and Technology; Sichuan Civil-military Integration Institute, Mianyang 621010, China

[4] Institute of Physics Nuclear and Chemistry, China Academy of Engineering Physics, Mianyang, 621900, China

[5] School of Physics and Electronic Information, Gannan Normal University, Ganzhou 341000, China

[6] Institute of Fluid Physics, China Academy of Engineering Physics, Mianyang 621900, China

*Corresponding author:

Zhengwei Xiong (zw-xiong@swust.edu.cn) and Zhipeng Gao (z.p.gao@foxmail.com)

[†] These authors contributed equally to this work.

By laser particle size analyzer, the average sizes of FeNiMo/SiO_2 powder cores with different TEOS volume can be obtained, as shown in Fig. S1. Obviously, the particle sizes are increasing with the increase of TEOS concentrations. Compared with the pure FeNiMo particles (Fig. 1b), the thickness of the SiO_2 layer can be deduced in the range of 900 nm-4.7 μm.

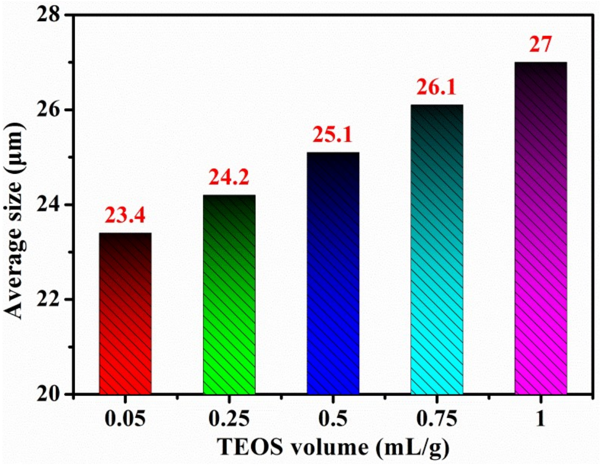

Fig. S1 Average sizes of FeNiMo/SiO_2 powder cores with different TEOS volume.

To confirm the core-shell structure, we further re-tested EDS elemental distribution mapping of FeNiMo/SiO_2 powders. According to select the incompletely coated particles (Fig. S2), we can clearly observe the formation of core-shell structures.

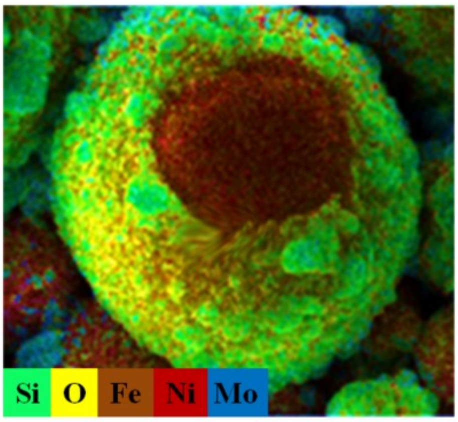

Fig. S2 EDS elemental distribution mapping of FeNiMo/SiO_2 powders.

Table S1. Elemental ratios of raw FeNiMo and FeNiMo/SiO_2 powders.

Element	Raw FeNiMo powders		FeNiMo/SiO_2 powders	
	wt.%	at.%	wt.%	at.%
Fe K	12.29	11.57	10.64	10.96
Ni	85.14	84.27	73.74	79.77
Mo	2.57	4.16	2.23	3.94
Si K	0	0	6.25	3.24
O K	0	0	7.14	2.10
Total	100		100	

Table S2. Comparison of magnetic properties with domestic and foreign commercial molypermalloy powder (MPP) cores.

Part Number	Company	Country	μ_{eff}	P_{cv} (kW/m^3,100 mT, 50 kHz)	P_{cv} (kW/m^3,100 mT, 100 kHz)	Data Sources
This work	-	China	78	263.29	633.44	-
Y60-102	Beijing Seven Star Flight Electron Co., Ltd	China	60	~600	-	[1]
KM050-060A	ZHEJIANG NBTM KEDA MAGNETOELECTRICITY CO., LTD (KDM)	China	60	250	-	[2]
C055041A2	Magnetics	USA	60	-	700	[3]
CM102060G	Chang Sung Corporation (CSC)	Korea	60	230	-	[4]

References

[1]http://798.com.cn/products1.aspx?ProductsCateId=32&CateID=32&CurrCateID=15&CurrsubCateID=32

[2] https://www.kda.com.cn/uploads/file/20211201/1638329222712889.pdf

[3] https://www.mag-inc.com/Media/Magnetics/Datasheets/C055041A2.pdf

[4]http://www.changsung.com/file/product/cores/3.%20MPP%20Cores/TDS-CM102060G.pdf
